# Deletion of the prorenin receptor in the ureteric bud in mice inhibits Dot1/H3K79 pathway

**DOI:** 10.1038/s41390-024-03026-5

**Published:** 2024-01-29

**Authors:** Renfang Song, Ihor V. Yosypiv

**Affiliations:** Section of Pediatric Nephrology, Department of Pediatrics, Tulane University Health Sciences Center, New Orleans, LA 70112 USA

## Abstract

**Background:**

The prorenin receptor (PRR) plays a critical role in ureteric bud (UB) branching morphogenesis. DOT1 Like (DOT1L), a histone methyltransferase specific for Histone 3 lysine 79 (H3K79), is important for differentiation of the UB-derived renal collecting duct cells. In this study, we tested whether DOT1L/H3 dimethyl K79 (H3m2K79) are regulated by *PRR* deletion in the UB and UB-derived collecting ducts in the embryonic mouse kidneys.

**Methods:**

Mutant *Hoxb7*^Cre+^/*PRR*^flox/flox^ (*PRR*^UB*-/-*^) and control *PRR*^UB+/+^, mice were studied on embryonic (E) day E17.5. DOT1L mRNA and protein expression in the kidney was examined by real-time qRT-PCR and immunohistochemistry, respectively. H3m2K79 protein expression was determined by immunohistochemistry and Western blot analysis.

**Results:**

*DOT1L* mRNA levels were decreased in mutant compared to control mice (0.68 ± 0.06 vs. 1.0 ± 0.01, *p* < 0.01). DOT1L and H3m2K79 immunostaining was reduced in the mutant vs. control kidneys (Dot1: 0.62 ± 0.03 vs. 1.0 ± 0.01, *p* < 0.05; H3m2K79: 0.64 ± 0.04 vs.1.1 ± 0.01. *p* < 0.05.). Western blot analysis revealed decreased H3m2K79 protein levels in mutant compared to control kidneys (1.0 ± 0.06 vs. 1.5 ± 0.02, *p* < 0.05).

**Conclusion:**

Targeted deletion of the *PRR* in the UB and UB-derived collecting ducts results in reduced *DOT1L* gene/protein and H3m2K79 protein expression in the embryonic mouse metanephroi in vivo.

**Impact:**

- The role of histone methylation in mediating the effect of the prorenin receptor on the ureteric bud branching (UB) morphogenesis and urine acidification during kidney development is unknown.
- We demonstrate that histone H3 lysine (K) 79 dimethylation by methyltransferase Dot1 is reduced in the embryonic kidney of mice that lack the prorenin receptor in the UB lineage.

## Introduction

Branching morphogenesis of the ureteric bud (UB) is a key developmental process that directs organogenesis of the kidney.^1,2^ Disruption of UB branching results in a variety of congenital anomalies of the kidney and urinary tract (CAKUT), including kidney agenesis (absence of the kidney), kidney hypoplasia (small kidney with reduced nephron number), hypodysplasia (small kidney with disorganized tissue histology), and ureter defects that can cause reflux of the urine or obstruction of the urine outflow.^2^ Collectively, CAKUT account for ~40% of end-stage kidney disease in children.^3^

We previously demonstrated that conditional ablation of the prorenin receptor (*PRR*) in the developing UB in mice (*PRR*^UB−/−^) using *HOXB7*^Cre^ driver reduces UB branching resulting in renal hypodysplasia, decreased nephron number at birth, polyuria and reduced ability to acidify the urine on postnatal (P) day P30.^4^ The PRR is the cell-surface receptor for renin and prorenin, and an accessory subunit of the vacuolar proton pump V-ATPase encoded for by *ATP6AP2* gene.^5^ In the adult rat collecting duct, PRR is most abundant at the apical surface of acid-secreting type α intercalated cells (α-ICs) where it colocalizes with the V-ATPase and may be activated in a paracrine fashion by prorenin or renin released by adjacent principal cells.^6^

Disruptor of Telomere Silencing (DOT1L) stimulates methylation of the histone mark H3K79 and generally indicates an active gene transcriptional state.^7^ DOT1L is expressed in the UB and collecting ducts in mice.^8^ Loss of DOT1L/H3K79me in principal cells of the UB-derived collecting ducts in mice results in increased number of acid-secreting intercalated cells and upregulates V-ATPase B1 subunit expression in these cells.^9^ In addition, DOT1L regulates *ATP6V1B1* gene transcription rate in kidney inner medullary collecting duct (IMCD3) cells in vitro.^9^ In this work, we tested the hypothesis that DOT1L/H3 dimethyl K79 (H3m2K79) are regulated by *PRR* deletion in the UB and UB-derived collecting ducts of the embryonic mouse kidneys.

## Methods

### Animals

All experiments involving mice were approved by Tulane Institutional Animal Care and Use Committee. To delete *PRR* conditionally in the UB, we used the *HOXB7*^Cre,GFP^ transgene, which drives Cre expression in the Wolffian duct and UB epithelium from E9.5 onward.^10^ The resulting *HOXB7*^Cre+,GFP+^/*PRR*^flox/flox^ mice represent UB-specific *PRR*-knockout mice (*PRR*^UB-/-^).^4^ Control mice consisted of *HOXB7*^Cre+,GFP+^/*PRR*^+/+^ (*PRR*^UB+/+^) littermates. Embryo sex was not determined.

### Isolation of embryonic kidneys

Embryonic kidneys were dissected from *PRR*^UB-/-^ and control mice on embryonic (E) day E17.5. Images were acquired using an Olympus IX70 inverted phase-contrast microscope.

### Quantitative reverse-transcription polymerase chain reaction (qRT-PCR)

qRT-PCR was performed in the Mx3000P equipment (Stratagene, La Jolla, CA) using MxPro QPCR software (Stratagene) as previously described.^4^ mRNA was extracted from snap-frozen E17.5 *PRR*^UB-/-^ and control kidneys [*n* = 3 mice (6 kidneys) per group]. Total mRNA was extracted and purified using QIAwave RNA Mini Kit (50),Cat. No. / ID: 74534(QIAGEN) according to the manufacture’s instruction. Reverse Transcription was performed using LunaScript RT SuperMix Kit (NEB #E3010)(New England Bio Labs) according to the manufacture’s protocol. The Taqman probes (Dot1 Mm01173481_m1 and GAPDH Mm99999915_g1) were ordered from Thermal Scientific for qRT-PCR assay. The quantity of DOT1 mRNA was normalized by that of GAPDH mRNA expression. RNA samples were analyzed in triplicates in each run. PCR reaction was performed twice.

### Immunohistochemistry

Kidneys from E17.5 *PRR*^UB-/-^ and control [*n* = 3 mice (6 kidneys) per group] were cut in the longitudinal midplane, processed through the paraffin, and embedded on the cut surface. Kidneys were sectioned at 4-µm and two consecutive sections adjacent to the longitudinal midplane were processed for immunostaining with each antibody (*n* = 12 sections per group). Immunostaining was performed by the immunofluorescent standard technique with Vectastain Elite kit (Vector Laboratories, Burlingame, CA). Primary antibodies included rabbit anti-DOT1L (Ab64077, Abcam, 1:1000), anti-H3 di-methyl K79 (Rabbit polyclonal, ab3594, Abcam), and anti-Aqp2 (goat anti-Aqp2; sc-9882, Santa Cruz, 1:100). Secondary antibodies were detected with Alexa Fluor dyes (Invitrogen). Specificity of immunostaining was documented by the omission of the primary antibody. The intensity of respective antibody immunofluorescence in kidney sections (*n* = 3 mice per group) was examined using Slide book 4.0 software (Intelligent Imaging Innovations, Denver, CO). All counts were performed in a blinded fashion.

### Western blot analysis

Kidneys from E17.5 *PRR*^UB–/–^ and control [*n* = 3 mice (6 kidneys) per group] were pooled and homogenized in cold lysis buffer containing a cocktail of enzyme inhibitors using RIPA Lysis and Extraction Buffer Catalog number: 89901 and Halt Protease Inhibitor Cocktail Part No. 78430 from Thermo Scientific™. The samples were centrifuged, and the supernatants containing proteins (30 μg/lane) were resolved on 4–12% gradient SDS-polyacrylamide gels and transferred to nitrocellulose membranes. After blocking nonspecific binding, the membranes were incubated with anti-H3 di-methyl K79 (Rabbit polyclonal, ab3594, Abcam) or anti-total histone H3 (1/1000, Upstate) antibodies at room temperature for 1 h. After washes in phosphate-buffered saline/Tween, the nitrocellulose membrane was exposed for 1 h at room temperature to the secondary antibody. Immunoreactive bands were visualized using the enhanced chemiluminescence detection system (ECL, Amersham, NJ).

### Statistical analysis

Statistical analyses were carried out upon all biologic replicates with Student’s *t* test or a one-way ANOVA. Data are presented as Mean ± SEM. A *p* value of <0.05 was considered statistically significant.

## Results

### Targeted deletion of the PRR in the UB lineage in PRR^UB-/-^ mice reduces DOT1L mRNA and protein expression in the embryonic kidney

**Fig. 1:**
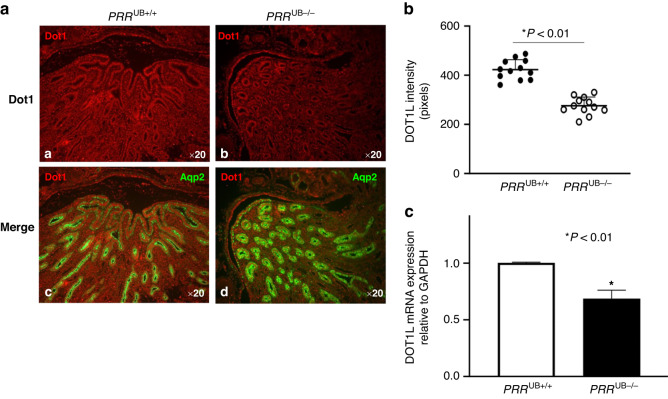
Targeted deletion of the *PRR* in the UB lineage in *PRR*^UB-/-^ mice reduces DOT1L mRNA and protein expression in the embryonic kidney. **a** a,b- DOT1L immunofluorescence is reduced in the papilla/medulla of the *PRR*^UB−/−^ compared to control mice; c,d- Double immunofluorescence staining shows Aqp2-positive principal cells of the collecting duct (green) and reduction in DOT1L immunofluorescence in the intercalated cells of the collecting duct (red) in mutant kidney. **b** Data point graph shows reduced DOT1L fluorescent intensity in mutant compared to control kidneys. **c** Bar graph shows reduced *DOT1L*/*GAPDH* mRNA ratios in mutant compared to control kidneys.

To determine whether lack of the *PRR* in the UB and UB-derived collecting ducts reduces expression of the histone H3K79 methyltransferase DOT1L, we examined DOT1L mRNA and protein expression in the kidney at E17.5. Kidney section surface area was smaller in the mutant compared to control mice (220600 ± 20120 vs. 533800 ± 72170 pixels, *p* < 0.05), consistent with renal hypoplasia observed in mutant mice in our previous study.^4^ (Fig. 1). In the embryonic kidney, besides abundant expression in the UB lineage, DOT1L is expressed in the cap mesenchyme, nephrons and at lower levels in cortical stromal cells.^8^ To determine the effect of *PRR* deficiency on DOT1L expression more specifically in the UB lineage, we quantitated DOT1L immunofluorescence intensity in the medulla and papilla areas where DOT1L staining should mainly represent expression in the UB/collecting ducts. DOT1L immunostaining, factored per kidney section surface area, was also reduced in the mutant vs. control kidneys (280 ± 35 vs. 400 ± 45 pixels, *p* < 0.01) (Fig. 1). *DOT1L* mRNA levels, factored per expression of the housekeeping gene *GAPDH*, were decreased in mutant compared to control mice (0.68 ± 0.06 vs. 1.0 ± 0.01, *p* < 0.01) (Fig. 1).

### Targeted deletion of the PRR in the UB lineage in PRR^UB–/–^ mice reduces H3m2K79 protein expression in the embryonic kidney

**Fig. 2:**
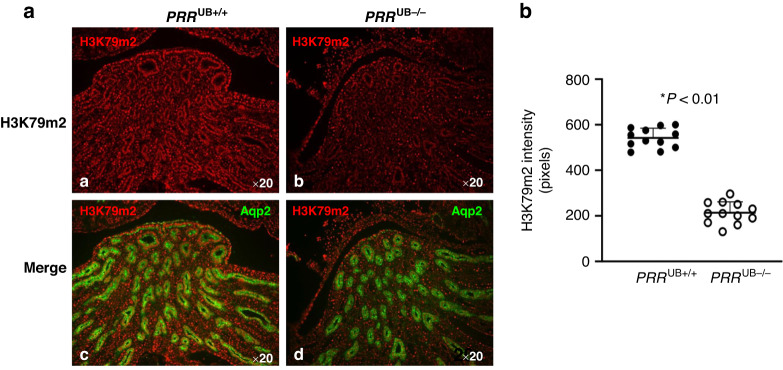
Targeted deletion of the *PRR* in the UB lineage in *PRR*^UB-/-^ mice reduces H3K79m2 protein expression in the embryonic kidney. **a** a,b- H3m2K79 immunofluorescence is reduced in the papilla/medulla of the *PRR*^UB−/−^ compared to control mice; c,d- Double immunofluorescence staining shows Aqp2-positive principal cells of the collecting duct (green) and reduction in H3K79m2 immunofluorescence in the intercalated cells of the collecting duct (red) in mutant kidney. **b** Data point graph shows reduced H3K79m2 fluorescent intensity in mutant compared to control kidneys.

**Fig. 3:**
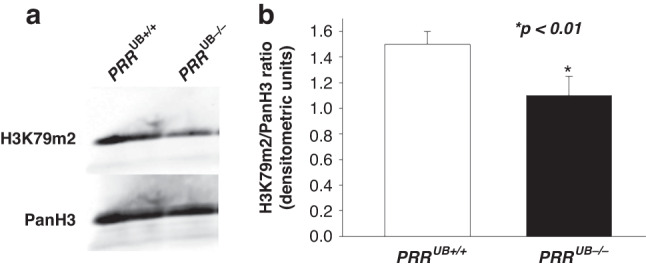
Targeted deletion of the *PRR* in the UB lineage in *PRR*^UB-/-^ mice reduces H3K79m2 protein expression in the embryonic kidney. **a** Western blot analysis shows reduction in the amount of H3K79m2 protein in *PRR*^UB–/–^ compared to control E17.5 kidneys. Pan histone H3 (PanH3) protein bands represent loading controls. **b** Bar graph shows reduced H3K79m2/PanH3 ratios in mutant compared to control kidneys.

To determine whether reduced expression of DOT1L *in PRR*^*UB-/-*^ kidneys is associated with decreased expression of its target H3K79m2, we measured the levels of H3K79m2 protein in *PRR*^*UB-/-*^ and control kidneys at E17.5 by immunofluorescence and Western blot analysis. H3K79m2 immunostaining, factored per kidney section surface area, was reduced in the medulla and papilla areas of the mutant vs. control kidneys (215 ± 46 vs. 550 ± 42 pixels, *p* < 0.01) (Fig. 2). Western blot analysis revealed decreased H3K79m2 protein levels in mutant compared to control kidneys (1.0 ± 0.06 vs. 1.5 ± 0.02 densitometric units, *p* < 0.05) (Fig. 3).

## Discussion

The present study demonstrates that targeted deletion of the *PRR* in the UB lineage in *PRR*^UB-/-^ mice reduces DOT1L mRNA and protein expression and decreases expression of its target H3K79m2 protein levels in the embryonic kidney.

Conditional ablation of the *PRR* in the developing UB in *PRR*^UB−/−^ mice inhibits UB branching, resulting in renal hypodysplasia and reduced ability to acidify the urine.^4^ Histone H3K79 methyltransferase DOT1L regulates differentiation of the UB-derived collecting duct cells during development in mice.^11^ In this study, we examined whether reduced UB branching and reduced urine acidification capacity in *PRR*^UB−/−^ mice is associated with a decrease in DOT1L/H3K79 methylation in the embryonic kidney.

Regulation of acid-base balance by the kidney is essential for health. In the collecting duct, α-intercalated cells (α-ICs) secrete protons into the tubular lumen through apical membrane V-ATPase.^12^ The PRR, encoded by *ATP6AP2*, is an accessory subunit of the V-ATPase.^5^ Differentiation of ICs from precursor UB cells requires the winged helix transcription factor FOXI1 as *FOXI1*^UB−/−^ mice develop distal renal tubular acidosis (dRTA) due to absence of ICs.^13^ *PRR* deletion in the collecting duct in *PRR*^UB−/−^ mice causes decreased expression of FOXI1, Cl^-^/HCO3^-^ exchanger (AE1) and α-IC-specific V-ATPase subunit α4.^4^ Thus, PRR is a direct or indirect regulator of gene expression in α-ICs during terminal differentiation of collecting duct cells. As mutations in genes encoding V-ATPase α4 subunit or AE1 result in genetic forms of dRTA in humans,^14^ the role of PRR in acid-base homeostasis may be clinically relevant.

Histone methylation regulates gene expression by controlling access to DNA-binding transcription factors.^15^ Disruptor of telomeric silencing (DOT1L) is methyltransferase specific for histone H3K79.^16^ DOT1L regulates gene transcription via histone methylation and global knockout of *DOT1L* results in embryonic lethality in mice.^17^ Targeted deletion of *DOT1L* in principal cells of the collecting duct in mice results in increased number of acid-secreting intercalated cells and upregulates V-ATPase B1 subunit expression in these cells most likely by increasing *ATP6V1V1* gene transcription rate.^9^

**Fig. 4:**
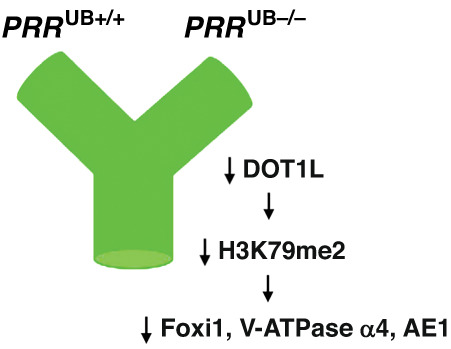
A working model depicting the role of the DOT1L/H3me2K79 in defective ureteric bud (UB) branching and intercalated cell differentiation in *PRR*^UB-/-^ mice. Deletion of *PRR* in the UB in *PRR*^UB-/-^ mice results in reduced DOT1L/H3K79me2 levels in the embryonic kidney. Decrease in DOT1L-mediated H3K79m2 may repress expression of genes promoting UB branching or genes promoting differentiation of UB cells into intercalated cell (IC) lineage (Foxi1). Decreased IC number may contribute, in part, to defects in urinary acidification capacity in *PRR*^UB-/-^ mice.

Our present findings of decreased DOT1L mRNA and protein expression in the embryonic *PRR*^UB-/-^ compared to control kidney, coupled with diminished expression of its target H3K79m2 protein levels, suggest that reduced H3K79m2 methylation may alter the expression of H3K79m2-target genes important for UB branching or differentiation of acid-secreting cells in the collecting duct. One possible mechanism how lack of UB *PRR* may decrease ability of the kidney to acidify the urine is by reduced methylation of *FOXI1* gene and its downstream targets, V-ATPase α4 and AE1 (Fig. 4). Here, DOT1L-mediated H3K79m2 may serve as a signal for repression of IC lineage during terminal differentiation of collecting duct cells.

In conclusion, targeted deletion of the *PRR* in the UB and UB-derived collecting ducts results in reduced *DOT1L* gene/protein and H3m2K79 protein expression in the embryonic mouse metanephroi in vivo. *DOT1L* mutations have not been described in human CAKUT. However, expression of many CAKUT-associated genes is altered in mice with targeted deletion of *DOT1L* in nephron progenitor lineage.^8^ Given that only 20–30% of human CAKUT cases are caused by known monogenic mutations, majority of CAKUT are likely due to undiscovered gene mutations or epigenetic modifications of gene expression. Future studies will determine whether H3K79 methylation downstream of the UB PRR plays a role in epigenetic regulation of collecting duct development and function.

## Data Availability

All data generated or analysed during this study are included in this published article.
